# *CKAP2L*, as an Independent Risk Factor, Closely Related to the Prognosis of Glioma

**DOI:** 10.1155/2021/5486131

**Published:** 2021-09-28

**Authors:** Li Zhu, Yanlei Zheng, Ronghua Hu, Chenchen Hu

**Affiliations:** Intensive Care Unit, Hubei Cancer Hospital, Hubei, China

## Abstract

Recent studies have found that cytoskeleton-associated protein 2 like (*CKAP2L*), an important oncogene, is involved in the biological behavior of many malignant tumors, but its function in the malignant course of glioma has not been confirmed. The main purpose of this study was to clarify the relationship between prognostic clinical characteristics of glioma patients and *CKAP2L* expression using data collected from the GEPIA, HPA, CGGA, TCGA, and GEO databases. *CKAP2L* expression was significantly increased in glioma. Further, Kaplan-Meier plots revealed that increased expression of *CKAP2L* was associated with shorter survival time of glioma patients in datasets retrieved from multiple databases. Cox regression analysis indicated that *CKAP2L* can serve as an independent risk factor but also has relatively reliable diagnostic value for the prognosis of glioma patients. The results of gene set enrichment analysis suggested that *CKAP2L* may play a regulatory role through the cell cycle, homologous recombination, and N-glycan biosynthesis cell signaling pathways. Several drugs with potential inhibitory effects on *CKAP2L* were identified in the CMap database that may have therapeutic effects on glioma. Finally, knockdown of CKAP2L inhibited the proliferation and invasion of cells by reducing the expression level of cell cycle-related proteins. This is the first study to demonstrate that high CKAP2L expression leads to poor prognosis in glioma patients, providing a novel target for diagnosis and treatment of glioma.

## 1. Introduction

Gliomas are primary malignant tumors derived from neural progenitor cells and glial cells that account for the vast majority of intracranial tumors [1, 2]. Because of the high degree of malignancy and invasive growth of glioma cells, gliomas lead to severe physiological dysfunction in patients and eventual death, reducing patient quality of life and increasing the burden on the healthcare system [3]. To improve the prognosis of patients with glioma, many scientists have made unremitting efforts to form the standardized therapeutic principles that have been developed to maximize tumor resection, reduce tumor load, and administer adjuvant radiotherapy and chemotherapy [4]. Despite these efforts, the prognosis of glioma patients has only partially improved, and survival rates remain poor [3]. One of the most important factors affecting the prognosis of glioma patients may be the lack of a sensitive and highly specific biomarker for targeted therapy.

During the process of tumorigenesis and development, a large number of tumor suppressor genes are known to be inactivated, whereas oncogenes are activated. The abnormal expression of these genes plays an extremely important role in regulating the growth of tumor cells, including gliomas [5]. With the development of molecular medicine and a deeper understanding of tumor pathological mechanisms at the molecular level, many of these abnormally expressed genes have been used as biomarkers in the diagnosis and treatment of tumors. In an effort to identify more reliable biomarkers, researchers have established a variety of publicly available databases, including TCGA and CGGA, with sufficient sample sizes and comprehensive clinical information. Mining this data provides guidance for molecular typing and drug target development for glioma, laying the foundation for development of a whole chain of precision medicine for glioma [6, 7]. To date, many effective biomarkers have been identified through database analysis methods for the diagnosis and targeted treatment of glioma, including CD276, HLA-F, and CMTM6 [8–10]. However, the complex pathological mechanisms of glioma may have resulted in many potential targets remaining unexplored, leading to lack of progress in improving the prognosis of glioma patients.

Cytoskeleton-associated protein 2 like (*CKAP2L*) plays an important regulatory role in the mitochondrial spin of neural progenitor cells. *CKAP2L* mutation is related to defects in the spindle tissue, including mitotic spindle defects, chromosome hysteresis, and other mitotic instability characteristics, that are involved in the formation and development of cancer [11]. Furthermore, a large number of previous studies have confirmed that *CKAP2L* is involved in the regulation of a variety of cancers. For example, Xiong et al. determined that abnormal expression of *CKAP2L* was highly correlated with poor prognosis of lung cancer and may be regulated by the MAPK signaling pathway [12]. Moreover, Wang and He reported that increased expression of *CKAP2L* in hepatocellular carcinoma (HCC) cells promoted their migration and invasion, which was associated with poor prognosis of HCC [13]. In addition, *CKAP2L* is reportedly involved in the occurrence and development of other malignant tumors, such as breast and prostate cancers, and is associated with poor prognosis of these cancers [14, 15]. However, it is worth noting that the relationship between *CKAP2L* and the prognostic features of gliomas has not been elucidated and revealed.

Therefore, this study was aimed at exploring the previously unknown relationship between *CKAP2L* and the biological characteristics of glioma patients, based on clinical sample analysis of multiple databases, and at confirming the oncogenic role of *CKAP2L* in glioma. We believe that the results of this study will provide a new target for the diagnosis and treatment of glioma, thus improving the prognosis of glioma patients.

## 2. Materials and Methods

### 2.1. Data Collection

Gene Expression Profiling Interactive Analysis (GEPIA; http://gepia.cancer-pku.cn/) is a public data platform containing gene expression levels and patient characteristics for the vast majority of human tumors [16]. Using this database, we collected *CKAP2L* expression data for 163 glioblastomas, 518 low-grade gliomas, and 207 normal brain tissue samples.

Gene Expression Omnibus (GEO) is a public repository of functional genomic data obtained via high-throughput and next-generation sequencing [17], which has been widely used for bioinformatics analysis of tumor characteristics. We retrieved three glioma microarray datasets from the GEO database. GSE4290, comprised mainly of American samples, contained 23 normal brain samples and 77 glioma samples. GSE50161, comprised mainly of American samples, contained 13 normal brain samples and 34 glioma samples. GSE116520, comprised mainly of Indian samples, contained eight normal brain samples and 34 glioma samples.

The Cancer Genome Atlas (TCGA; http://cancergenome.nih.gov/) is an internationally renowned public data platform containing large-scale genome sequencing analysis of more than 30 human tumors from mainly white and black races [18] and has conducted large-scale genome sequencing analysis, which contains the main white and black races in the database. We obtained mRNA sequences and clinical information for 653 patients with glioma from TCGA database (Table S1).

The Chinese Glioma Genome Atlas (CGGA) is a public data platform focusing on brain tumor research that contains various types of data for more than 2000 tissue samples from Chinese patients. Excluding samples with incomplete clinical information, we obtained an RNA-seq dataset of 748 glioma samples and RNA-microarray data from 268 glioma samples (Table S2 and Table S3) [19].

According to the 2016 World Health Organization Classification of Tumors of the Central Nervous System, we obtained 40 glioma tissue samples and 5 nontumor brain tissue samples from patients at the Hubei Cancer Hospital, including 17 cases of grade III glioma and 23 cases of grade IV glioma. The clinical and molecular characteristics of the 40 glioma patients and their corresponding follow-up characteristics were collected (Table S4). The tissue samples were stored at 80°C for total RNA extraction. The study protocol was performed according to the Declaration of Helsinki and was approved by the Ethics Committee of Hubei Cancer Hospital.

### 2.2. Gene Set Enrichment Analysis for *CKAP2L*

Gene set enrichment analysis (GSEA) is a commonly used biological information tool [20] that can simultaneously analyze the expression profiles of multiple genes, enabling accurate identification of a target gene's function mechanism. In this study, we divided the data obtained from TCGA and CGGA databases into two groups according to expression levels of *CKAP2L*. GSEA 4.0. jar software was employed to analyze the cell signaling pathways associated with *CKAP2L*. KEGG pathways were selected after 1000 iterations.

### 2.3. Cell Culture

Normal human astrocytes and glioma cell lines LN229, T98, U251, and A172 were purchased from Procell Life Science & Technology Co, Ltd. (Hyderabad, India). All cells were incubated in Dulbecco's modified Eagle's medium (Invitrogen, Carlsbad, CA, USA) supplemented with 10% fetal bovine serum (Hyclone, Logan, UT, USA) at 37°C under 5% CO_2_ in a humidified incubator. Expression of *CKAP2L* was measured in all five cell lines, as described below, and the cell line with the highest *CKAP2L* expression was selected for further experiments. Accordingly, U251 cells were transfected with lentiviruses containing control shRNA or shRNA against *CKAP2L* (5′-GATCCGCAAACAAAGAGAACTTGCTCGATATTTCAAGAGAATATCGAGCAAGTTCTCTTTGTTTGTTTTTTGGAAG-3′), which were purchased from GenePharma (Suzhou, China), and screened using puromycin.

### 2.4. RT-qPCR Analysis

A Total RNA Kit I (Omega Bio-tek, Norcross, GA, USA) was used to extract the total RNA from cells and brain samples, and a NanoDrop spectrophotometer (Thermo Fisher Scientific, Waltham, MA, USA) was used to confirm the mass of total extracted RNA. Then, cDNA was reverse transcribed from total RNA using NovoScript Plus All-in-one 1st Strand cDNA Synthesis SuperMix (Novoprotein, Shanghai, China). The expression of *CKAP2L* was analyzed using RT-qPCR. The primer sequences were as follows: forward 5′-GGGAAAACTGAAGAGCCAAAACA-3′ and reverse 5′-AGGTTTGACAGGCAAAACAACA-3′. Expression levels were calculated using the 2^−*ΔΔ*CT^ method.

### 2.5. MTT Assay

U251 cells were plated at a density of 5 × 10^3^ cells/well in 96-well cell culture plates after transfection with shRNA for 24 h. Cells were treated with 20 *μ*L MTT reagent (5 mg/mL) at selected times (0, 24, 48, 72, and 96 h) and then incubated for 4 h. Subsequently, 150 *μ*L DMSO was used to dissolve the formazan crystals in each well, followed by incubation for 15 min. Finally, the absorbance was measured at 490 nm using a microplate reader.

### 2.6. Wound Healing Assay

U251 cells were plated at a density of 2 × 10^5^ cells/well in 6-well cell culture plates after transfection with shRNA for 24 h. After incubation for another 24 h at 37°C under 5% CO_2_, the U251 cells formed a monolayer, which was linearly scratched with a sterile pipette tip. The detached cells were washed off, and serum-free cell culture medium was added to the treated wells. Cell migration into the wound was measured after incubation for 24 h, and the distance was calculated using ImageJ software (National Institutes of Health, Bethesda, MD, USA).

### 2.7. Western Blotting

Total protein was extracted from U251 cells treated with shRNA, the proteins were then fractionated by 12% SDS-PAGE and transferred onto a PVDF membrane, and the membrane was blocked with 5% nonfat milk. Subsequently, the membrane was incubated with primary specific antibodies for cell cycle-related proteins, including CDK4 (1 : 1000, Proteintech, Chicago, IL, USA), CCNB1 (1 : 1000, Proteintech), CCND1 (1 : 1000, Proteintech), CCNE1 (1 : 1000, Proteintech), and GAPDH (1 : 1000, Proteintech), followed by three washes with TBST for 5 min. Then, horseradish peroxidase-conjugated secondary antibodies were applied for 1 h at room temperature, followed by three washes with TBST for 10 min. Finally, enhanced chemiluminescence was used to detect the cell cycle-related proteins, and signals were captured by Image Lab software.

### 2.8. CMap

The Connectivity Map (CMap; https://portals.broadinstitute.org/cmap/) database is used to compare changes in gene expression profiles of cultured human cells treated with small-molecule drugs to enable prediction of drugs to treat target diseases [21]. We used Pearson correlation coefficients to obtain the top 10 genes positively (upregulated) and negatively (downregulated) correlated with *CKAP2L*, based on the RNA-seq dataset of 748 glioma samples obtained from the CGGA database. Potential small-molecule drugs with an inhibitory effect on *CKAP2L* were identified in CMap when *P* < 0.05 and enrichment < −0.8. Finally, the 3D and 2D structures of these drugs and their molecular formulas were obtained from the PubChem database (https://pubchem.ncbi.nlm.nih.gov/) [22].

### 2.9. Statistical Analysis

All data were statistically analyzed using R language software (version 3.6.1). The comparison of *CKAP2L* expression in tumor and normal brain tissues was performed using the limma package in R software according to the cut-off standard (*P* < 0.05 and log FC > 1). Unpaired *t*-tests were applied to analyze the difference in *CKAP2L* expression between the two groups, with *P* < 0.05 considered significant. Kaplan Meier curves and Cox regression analysis (univariate and multivariate) were used to determine the impact of *CKAP2L* on the prognosis and diagnostic value of glioma patients. Pearson correlation coefficients were used to identify genes that were coexpressed with *CKAP2L*. *P* values < 0.05 were considered statistically significant.

## 3. Results

### 3.1. CKAP2L Expression Correlated with Overall Survival in Glioma

**Figure 1:**
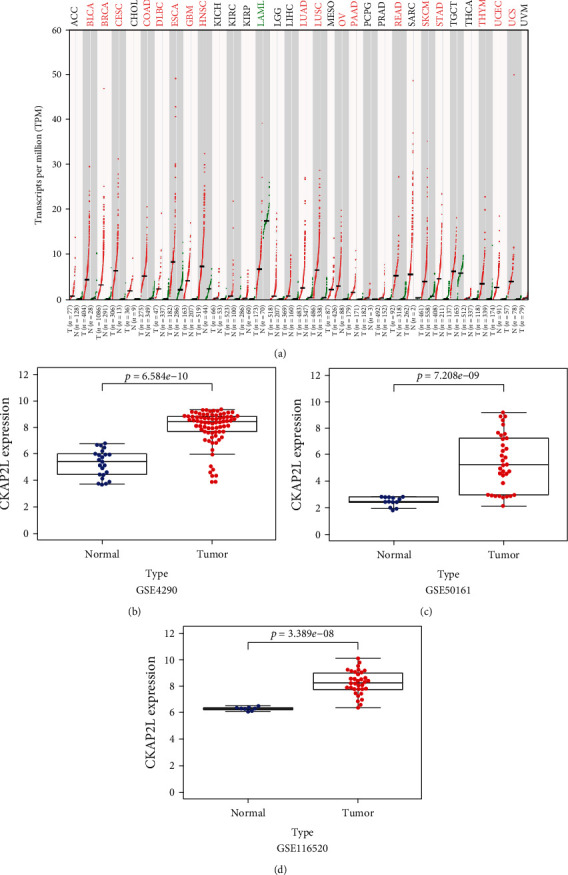
The expression of *CKAP2L* mRNA in glioma tissues was compared with that in normal brain tissues. (a) *CKAP2L* expression in various cancers in the GEPIA database: red indicates high expression of *CKAP2L* in the tumor tissue, and green indicates low expression. (b–d) The expression of *CKAP2L* in gliomas was increased in GSE4290, GSE50161, and GSE116520, respectively.

GEPIA database analysis revealed abnormal *CKAP2L* expression in human malignancies. The expression level of *CKAP2L* in acute myeloid leukemia (LAML) was significantly lower than that in the normal control group, but expression of *CKAP2L* in most malignant tumors was abnormally increased, including in glioblastoma (GBM), as shown in Figure 1(a). Analysis of three different glioma-related datasets in the GEO database, including dozens of normal brain samples and hundreds of glioma samples, revealed that the expression level of *CKAP2L* in glioma tissues was significantly higher (*P* < 0.05) than that in normal brain tissue, as shown in Figures 1(b)–1(d).

**Figure 2:**
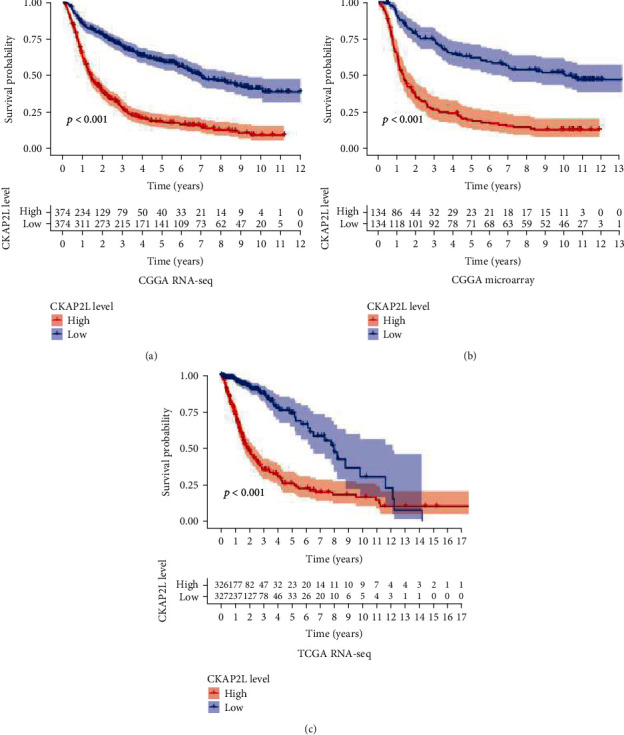
The relationship between *CKAP2L* and the prognosis of glioma patients in the CGGA and TCGA databases: (a) in CGGA RNA-seq; (b) in CGGA RNA-microarray; (c) in TCGA RNA-seq.

To further study the relationship between *CKAP2L* expression and prognosis of glioma, we analyzed three transcriptome datasets: CGGA microarray, CGGA RNA-seq, and TCGA RNA-seq. As shown in Figures 2(a)–2(c), increased expression of *CKAP2L* in the three datasets was associated with decreased patient overall survival (*P* < 0.001). The results were consistent in thousands of tissue samples, reliably indicating that high expression levels of *CKAP2L* led to poor prognosis in glioma patients.

**Figure 3:**
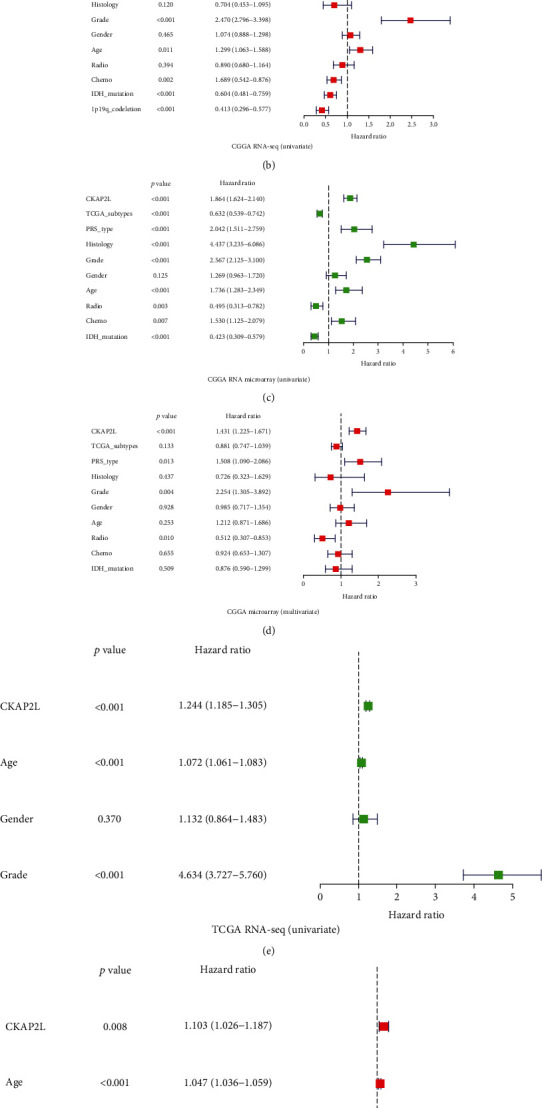
*CKAP2L* can be used as an independent risk factor for glioma: (a, b) in CGGA RNA-seq; (c, d) in CGGA RNA-microarray; (e, f) in TCGA RNA-seq.

**Figure 4:**
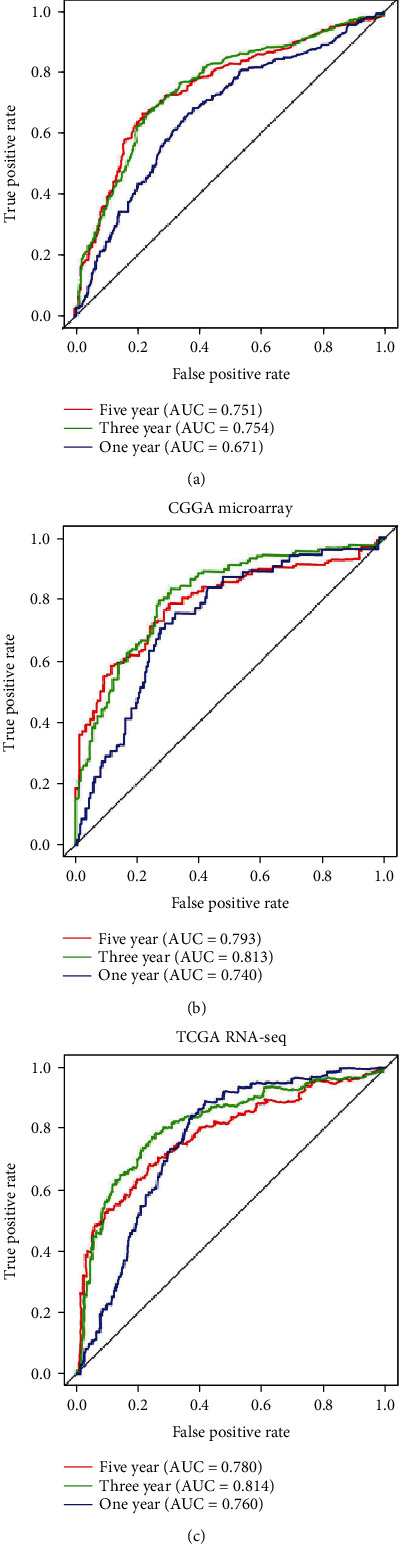
*CKAP2L* has diagnostic value for glioma in the CGGA and TCGA databases: (a) in CGGA RNA-seq; (b) in CGGA RNA-microarray; (c) in TCGA RNA-seq.

Receiver operating characteristic (ROC) curve analysis was employed to determine whether *CKAP2L* has diagnostic value in the prognosis of glioma patients (Figure 3). The area under the ROC curve for one, three, and five years using data obtained from the three transcriptome datasets was >0.7, indicating that *CKAP2L* has moderate diagnostic value, excluding the CGGA RNA-seq dataset for 1 year (Figures 4(a)–4(c)). These results supported that *CKAP2L* has a diagnostic value for glioma patients with poor prognosis.

### 3.2. *CKAP2L* Can Be an Independent Risk Factor of Glioma Patients

To investigate whether *CKAP2L* can be used as an independent risk factor for poor prognosis of glioma, we analyzed the three transcriptome datasets by univariate and multivariate Cox analyses. The univariate Cox analysis results indicated that increased *CKAP2L* expression could be a risk factor for poor prognosis in glioma patients in the CGGA RNA-seq (HR = 1.796; 95%CI = 1.636-1.973, *P* < 0.001), CGGA microarray (HR = 1.864; 95%CI = 1.624-2.140, *P* < 0.001), and TCGA RNA-seq (HR = 1.244; 95%CI = 1.185-1.305, *P* < 0.001) datasets. Increased tumor grade in patients with glioma was also a risk factor for poor prognosis in the CGGA RNA-seq (HR = 2.883; 95% CI = 2.526-3.291, *P* < 0.001), CGGA microarray (HR = 2.567; 95%CI = 2.125-3.100, *P* < 0.001), and TCGA RNA-seq (HR = 4.634; 95%CI = 3.727-5.760, *P* < 0.001) datasets. Furthermore, age was also a risk factor for poor prognosis in glioma patients in the CGGA RNA-seq (HR = 1.624; 95%CI = 1.345-1.960, *P* < 0.001), CGGA microarray (HR = 1.736; 95%CI = 1.283-2.349, *P* < 0.001), and TCGA RNA-seq (HR = 1.072; 95%CI = 1.061-1.083, *P* < 0.001) datasets (Figures 3(a), 3(c), and 3(e)).

At the same time, multivariate Cox analysis revealed that increased expression of *CKAP2L* could be an independent risk factor for poor prognosis in glioma patients in the CGGA RNA-seq (HR = 1.282; 95%CI = 1.148-1.431, *P* < 0.001), CGGA microarray (HR = 1.431; 95%CI = 1.225-1.671, *P* < 0.001), and TCGA RNA-seq (HR = 1.103; 95%CI = 1.0.26-1.187, *P* < 0.001) datasets. Tumor grade in the CGGA RNA-seq (HR = 2.470; 95%CI = 1.796-3.398, *P* < 0.001), CGGA microarray (HR = 2.254; 95%CI = 1.305-3.892, *P* < 0.001), and TCGA RNA-seq (HR = 2.918; 95%CI = 2.289-3.721, *P* < 0.001) datasets was found to be an independent risk factor for glioma patients (Figures 3(b), 3(d), and 3(f)). Further, the PRS type was an independent risk factor for poor prognosis of glioma patients in the CGGA RNA-seq (HR = 1.935; 95%CI = 1.646-2.275, *P* < 0.001) and CGGA microarray (HR = 1.508; 95%CI = 1.090-2.086, *P* < 0.001) datasets, as shown in Figures 3(b) and 3(d). The above results indicated that *CKAP2L* can be an independent risk factor associated with poor prognosis of patients with glioma.

### 3.3. GSEA Predicts Signaling Pathways Associated with *CKAP2L* and Glioma

**Figure 5:**
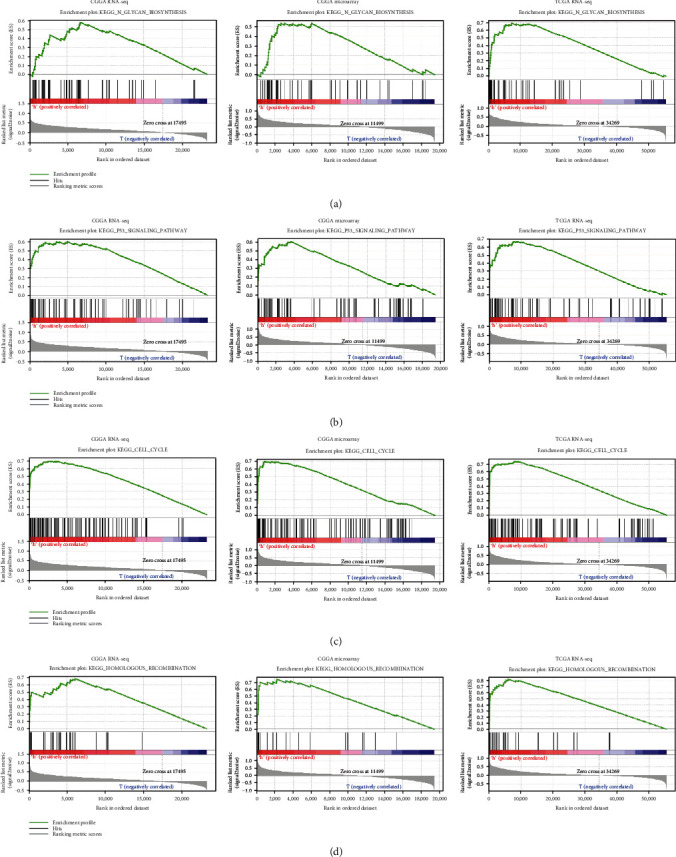
GSEA predicts signal pathways related to *CKAP2L* in TCGA and CGGA database: (a) N-glycan biosynthesis; (b) P53 signaling pathway; (c) cell cycle; (d) homologous recombination.

GSEA was used to analyze the potential cellular signaling pathways associated with *CKAP2L*. Analysis of three transcriptome datasets revealed that increased expression of *CKAP2L* can lead to activation of the p53, cell cycle, homologous recombination, and N-glycan biosynthesis cell signaling pathways (Figure 5 and Table 1). These results indicated that *CKAP2L* may regulate the occurrence and development of glioma through the above cell signaling pathways.

### 3.4. CKAP2L Expression Correlated with Tumor Grade

**Figure 6:**
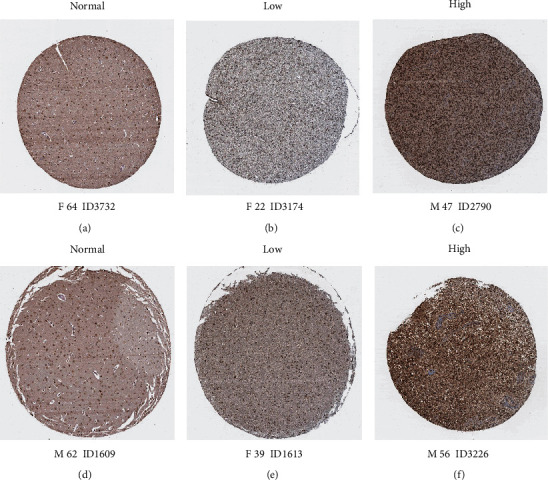
The expression of *CKAP2L* in normal brain tissue and glioma tissue: (a, d) normal brain tissue; (b, e) low-grade glioma tissue; (c, f) high-grade glioma tissue.

To further verify the *CKAP2L* expression profile in glioma, we examined CKAP2L protein levels in glioma data from The Human Protein Atlas, revealing markedly higher CKAP2L protein levels in low-grade and high-grade glioma tissues than in normal brain tissue (Figure 6).

### 3.5. Validation of Carcinogenic Effect of CKA*P2L*

**Figure 7:**
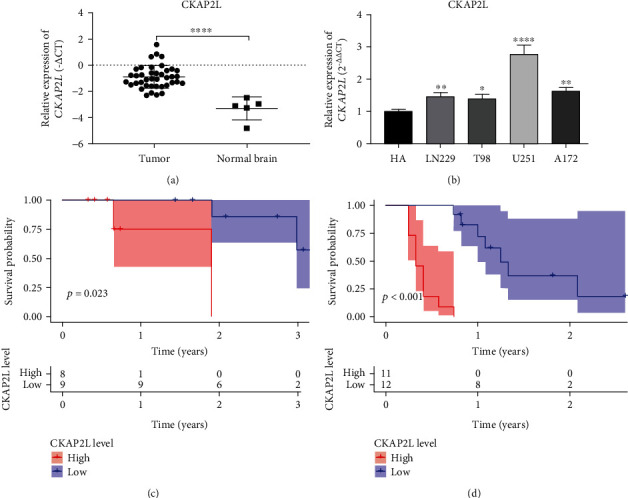
*CKAP2L* expression and clinical effect on glioma patients: (a) *CKAP2L* expression in normal brain tissues and glioma tissues; (b) *CKAP2L* expression in different glioma cell lines; (c) the survival curve of high *CKAP2L* expression in low-grade glioma patients; (d) the survival curve of high *CKAP2L* expression in high-grade glioma patients.

We further validated the influence of *CKAP2L* on patients with glioma by analyzing 40 clinical samples, consisting of both high-and low-grade glioma tissues, and the patient characteristics and outcomes. *CKAP2L* expression in glioma was significantly increased compared with that in normal brain tissue (Figure 7(a)). Further, high expression of *CKAP2L* led to shorter survival times for both high-and low-grade glioma patients (Figures 7(c) and 7(d)). Moreover, we examined the expression of *CKAP2L* in normal human astrocytes and four different glioma cell lines, determining the highest CKAP2L expression in U251 cells (Figure 7(b)). Therefore, we verified that the increased expression level of CKAP2L in glioma in the database resulted in the decrease of overall survival time of glioma patients. Based on the highest expression level of CKAP2L of U251 among the four glioma cell lines, we chose U251 cells to perform subsequent in vitro experiments.

**Figure 8:**
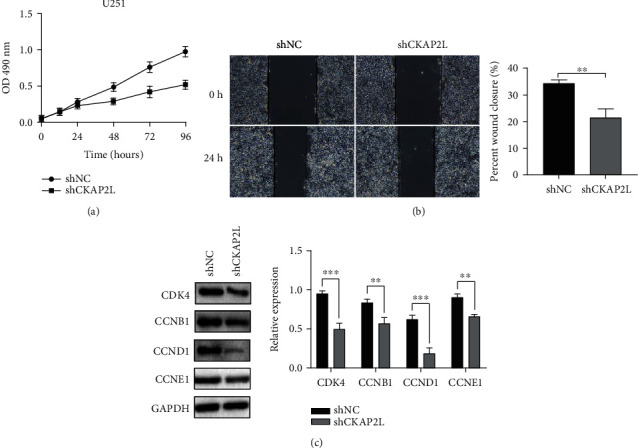
*CKAP2L* affected the biological characteristics of U251 cells in vitro: (a) knockdown of *CKAP2L* inhibited the proliferation of U251 cells; (b) knockdown of *CKAP2L* reduced the migration ability of U251 cells; (c) knockdown of *CKAP2L* downregulated the expression of cell cycle-related genes.

Using shRNA to knock down the expression of *CKAP2L*, we then performed MTT and wound healing assays to investigate the effect of *CKAP2L* on the biological characteristics of glioma cells. As shown in Figures 8(a) and 8(b), downregulation of *CKAP2L* remarkably inhibited the proliferation and migration of U251 cells. Furthermore, knockdown of *CKAP2L* significantly reduced the expression of cell cycle-related genes (Figure 8(c)). Therefore, the high expression of CKAP2L may promote the proliferation of glioma cells through the activation of cell cycle signal pathway, which has been further verified.

### 3.6. Gene Coexpression with *CKAP2L* and Drugs Targeting *CKAP2L*

**Figure 9:**
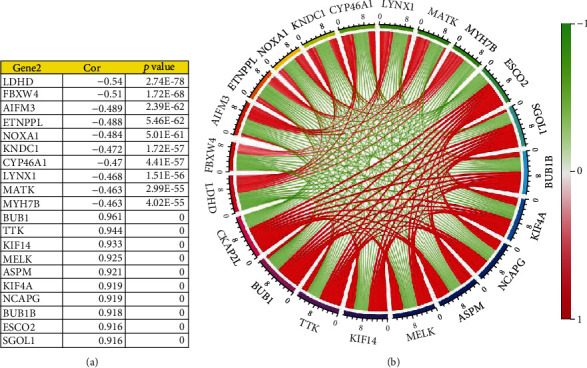
Genes related to *CKAP2L*.

**Figure 10:**
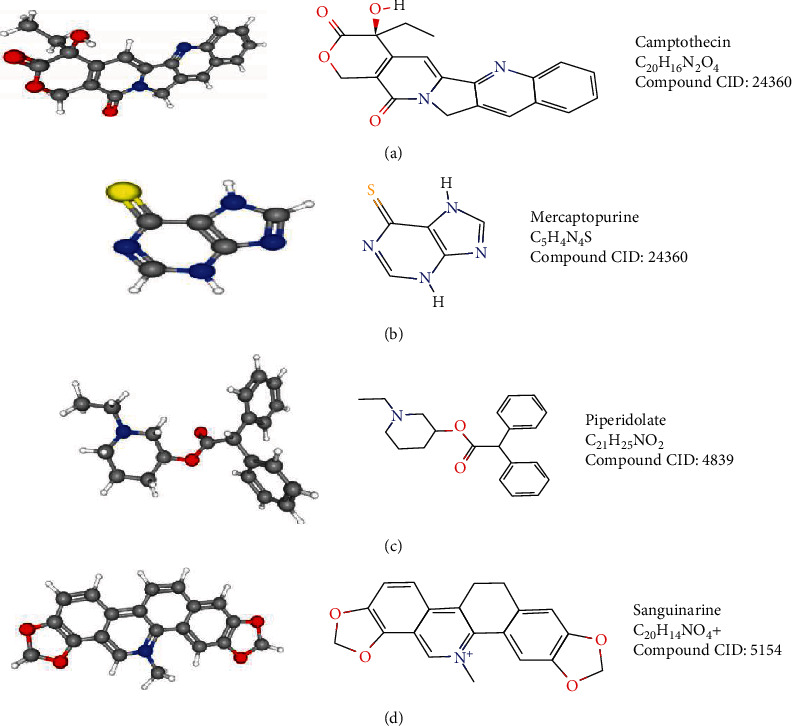
Drugs that potentially inhibit *CKAP2L*: (a) camptothecin; (b) mercaptopurine; (c) piperidolate; (d) sanguinarine.

Applying Pearson correlation analysis to the RNA-seq dataset of 748 glioma samples obtained from the CGGA database, we identified 10 genes positively correlated with *CKAP2L* (*BUB1*, *TTK*, *KIF14*, *MELK*, *ASPM*, *KIF4A*, *NCAPG*, *BUB1B*, *ESCO2*, and *SGOL1*) and 10 genes negatively correlated with *CKAP2L* (*LDHD*, *FBXW4*, *AIFM3*, *ETNPPL*, *NOXA1*, *KNDC1*, *CYP46A1*, *LYNX1*, *MATK*, and *MYH7B*) (Figure 9). Genes positively and negatively correlated with *CKAP2L* were regarded as upregulated and downregulated, respectively. After screening the genes in the CMap database to find drugs with targeted inhibitory effects on *CKAP2L*, four potentially therapeutic drugs were identified based on the search criteria, including camptothecin, mercaptopurine, piperidolate, and sanguinarine (Table 2). Finally, we obtained the 2D and 3D structures and molecular formulas of these drugs from PubChem, as shown in Figure 10.

## 4. Discussion

A large body of literature has confirmed that *CKAP2L* is involved in regulating many types of malignant tumors, promoting the proliferation of tumor cells, and shortening the survival time of patients. For example, Xu et al. reported that *CKAP2L* is involved in the biological behavior of prostate cancer and has a significant relationship with its malignant clinical features [15]. Similar conclusions have been reached for other malignant diseases such as hepatocellular carcinoma and lung cancer [12, 13]. However, to date, *CKAP2L* has not been reportedly associated with glioma.

To determine whether *CKAP2L* expression differs in glioma, we first investigated the expression of *CKAP2L* in glioma-related datasets obtained from GEPIA, HPA, and GEO. The results demonstrated that *CKAP2L* expression was higher in glioma tissues than in normal brain tissues at both the mRNA (Figure 1) and protein (Figure 6) levels. We employed a variety of data fusion analyses, based on thousands of multiethnic tissue samples to improve the scientific rigor and credibility of our results, and further verified the expression of *CKAP2L* in collected clinical samples and commercial glioma cell lines (Figure 7).

To confirm the impact of abnormally high expression of *CKAP2L* on the prognosis of glioma patients, we collected patient information from three different TCGA and CGGA transcriptome datasets and 40 clinical glioma patients. Kaplan-Meier curves demonstrated that increased *CKAP2L* expression was closely associated with poor prognosis of glioma patients. Concurring with our results, studies with liver, prostate, and lung cancers have reported that high expression of *CKAP2L* is associated with poor prognosis in tumor patients and can be used as a marker for diagnosis and treatment [13–15, 23]. Besides, the clinical molecular characteristics of glioma are closely related to the prognosis of patients. For example, some studies suggest that with the increase of glioma tumor grade, the heterogeneity of tumor cells also increases, and it has a negative correlation with the prognosis of patients [24]. Secondly, due to the infiltrative growth of glioma cells, the recurrence and secondary of glioma are the most important reasons leading to poor prognosis [25] and the incidence rate and mortality of glioma are positively correlated with the age of the patients [24]. Finally, molecular characteristics are closely related to the prognosis of gliomas, especially 1p19q codeletion and IDH mutation which are a good indicator for the prognosis of gliomas [26]. More importantly, in the WHO glioma classification in 2016, these two molecules have been used as a reference indicator of the malignant degree of glioma, and their different states will lead to a greater difference in the prognosis of patients [27]. Therefore, the above reports are consistent with the results of our multivariate analysis, which show that grade, recurrence, secondary, and age were risk factors for the prognosis of patients with glioma, and 1p19q codeletion and IDH mutation had a protective effect on the prognosis of patients. In addition to the above clinical and molecular characteristics of glioma patients, the status of radiotherapy and chemotherapy in our univariate analysis tends to be a risk factor, but the multivariate analysis is actually a protective factor for the prognosis of patients. We speculate that the prognosis of patients with low-grade gliomas is better, but they generally do not always receive chemoradiotherapy. However, patients with glioblastoma generally use chemoradiotherapy, so there is a risk factor in univariate analysis. Therefore, in the results of univariate analysis, the chemoradiotherapy group showed a risk factor. Our multivariate analysis is consistent with previous literature reports, which shows that the prognosis of glioma patients treated with radiotherapy and chemotherapy can improve the prognosis of patients [28]. Therefore, our study findings support that *CKAP2L* is associated with poor prognosis of glioma, can be used as an independent risk factor, and has diagnostic value.

Although the results of this current study confirmed that *CKAP2L* is a novel oncogenic gene that significantly reduces the survival time of patients, the molecular mechanism underlying the biological function of *CKAP2L* remains unknown. GSEA is a scientific, reliable, and widely used method in the biomedical field [19]. Here, GSEA of the above three datasets suggested that *CKAP2L* may participate in the p53, cell cycle, homologous recombination, and N-glycan biosynthesis signaling pathways. p53 is known to regulate a variety of signaling pathways, including the tumor cell cycle, autophagy, apoptosis, and aging. For example, Zhang et al. demonstrated that p53 can regulate the cell cycle to inhibit pancreatic cancer cell proliferation [29]. Research on the role of N-glycan in tumors has recently attracted increasing attention. N-Glycan can reportedly be used as a diagnostic marker for germline stem cell tumors and is associated with poor prognosis of patients with tumors [30]. Further, studies have reported that blocking N-polysaccharide precursor biosynthesis can inhibit the growth of U87 glioblastoma cells and significantly inhibit glioma size *in vivo* [31]. GSEA in the current study indicated that *CKAP2L* may be involved in the abovementioned cell signaling pathways and our subsequent *in vitro* experiments determined that knockdown of *CKAP2L* decreased the expression of key cell cycle-related proteins. The MTT and wound healing assays further demonstrated that *CKAP2L* influenced the proliferative and migratory abilities of glioma cells.

Through gene correlation analysis, we identified genes positively correlated with *CKAP2L*, including *BUB1*, *TTK*, and *ASPM*, which can promote the development of glioma and treatment resistance. These genes also indirectly support the hypothesis that *CKAP2L* may promote the pathological process of glioma. For example, *BUB1* is a mitotic checkpoint that can promote the proliferation of glioma cells and radioactive resistance and is directly associated with poor prognosis of glioma patients [32]. *TTK* reportedly plays a role in promoting the growth of gliomas. Indeed, silencing *TTK* can inhibit the proliferation, invasion, and radiation resistance of glioma cells and other malignant behaviors [33]. CMap analysis predicted four drugs that could negatively regulate *CKAP2L*. The therapeutic effects of two of these drugs on glioma have been previously reported, including camptothecin, a nanoprodrug with inhibitory effects on U87-MG glioma cell proliferation [34], and sanguinarine, which can reportedly induce ROS-dependent ERK1/2 activation and autophagy cell death in glioma cells [35]. The above findings support that *CKAP2L* may act synergistically with these identified genes, which may lead to poor prognosis of glioma patients, and the drugs identified via CMap may potentially inhibit *CKAP2L* and treat glioma.

Although we made every effort to elucidate the relationship between *CKAP2L* and the prognosis of glioma patients, the use of public databases has certain limitations, such as inconsistent clinical information across different databases. Therefore, our analysis does not include all clinical treatment details and individualized efficacy. However, we must emphasize that public databases also enable access to sufficient numbers of research samples in a short period of time and encompass a variety of ethnicities, which would be difficult to establish within a clinical study. Therefore, the reliability and authenticity of our analysis results were greatly improved through the use of multiple public databases.

## 5. Conclusions

The study results demonstrate that *CKAP2L* expression increases with the grade of glioma and is associated with poor prognosis of glioma patients. GSEA revealed that *CKAP2L* can regulate the development of glioma cells through various signaling pathways, especially that of the cell cycle. Besides, knockdown of CKAP2L inhibited the proliferation and invasion of cells by reducing the expression level of cell cycle-related proteins. Our findings elucidate the possible role of CKAP2L in glioma, identifying a reliable biological biomarker for its diagnosis and treatment.

## Figures and Tables

**Table 1:** Cell signaling pathway that *CKAP2L* may be enriched.

Gene set name	CGGA RNA-seq			CGGA -microarray			TCGA RNA-seq		
	NES	NOM P *P* val	FDR *q*-val	NES	NOM P *P* val	FDR *q*-val	NES	NOM *P* val	FDR *q*-val
Cell cycle	2.022	0.000	0.014	2.132	0.000	0.003	2.251	0.000	0.000
P53 signaling pathway	1.833	0.000	0.069	2.038	0.000	0.007	2.199	0.000	0.001
Homologous recombination	1.813	0.008	0.065	1.985	0.000	0.017	2.079	0.000	0.003
N-Glycan biosynthesis	1.698	0.006	0.132	1.655	0.043	0.155	2.005	0.002	0.007

NES: normalized enrichment score; NOM: nominal; FDR: false discovery rate. Gene sets with NOM *P* value < 0.05 and FDR *q* − value < 0.25 were considered as significantly enriched.

**Table 2:** Screened drugs from CMap.

No.	CMap name	Enrichment	*P*
1	Camptothecin	-0.881	0.00335
2	Piperidolate	-0.841	0.00801
3	Sanguinarine	-0.849	0.04565
4	Mercaptopurine	-0.846	0.04785

Enrichment < −0.8，*P* < 0.05; CMap: connectivity map.

## Data Availability

All data generated or analyzed during this study are included in this published article and its supplementary information files.

## Supplementary Materials

Supplementary 1 Table S1: characteristics of patients with glioma based on TCGA.

Supplementary 2 Table S2: characteristics of patients with glioma based on CGGA.

Supplementary 3 Table S3: characteristics of patients with glioma based on CGGA microarray.

Supplementary 4 Table S4: characteristics of patients with glioma based on clinical patients.
