# Supplementary material for: CKAP2L, as an Independent Risk Factor, Closely Related to the Prognosis of Glioma
Source: Biomed Res Int. 2021 Sep 28;2021:5486131. doi: 10.1155/2021/5486131 (PMC8494202; doi:10.1155/2021/5486131)
Supplement: Supplementary 1 — Table S1: characteristics of patients with glioma based on TCGA. [file 5486131.f1.docx]

Table S1. Characteristics of patients with glioma based on TCGA

| Characteristics |  | Number of cases | Percentages(%) |
| --- | --- | --- | --- |
| Gender | Male | 380 | 57.84 |
|  | Female | 277 | 42.16 |
| Age | <=51 | 397 | 60.43 |
|  | >51 | 260 | 39.57 |
| Grade | WHO II | 240 | 36.53 |
|  | WHO III | 257 | 39.12 |
|  | WHO IV | 160 | 24.35 |
