# Supplementary material for: CKAP2L, as an Independent Risk Factor, Closely Related to the Prognosis of Glioma
Source: Biomed Res Int. 2021 Sep 28;2021:5486131. doi: 10.1155/2021/5486131 (PMC8494202; doi:10.1155/2021/5486131)
Supplement: Supplementary 2 — Table S2: characteristics of patients with glioma based on CGGA. [file 5486131.f2.docx]

Table S2. Characteristics of patients with glioma based on CGGA

| Characteristics |  | Number of cases | Percentages(%) |
| --- | --- | --- | --- |
| Gender | Male | 307 | 40.99 |
|  | Female | 442 | 59.01 |
| Age | <=41 | 342 | 45.66 |
|  | >41 | 407 | 54.34 |
| Grade | WHO II | 218 | 29.11 |
|  | WHO III | 240 | 32.04 |
|  | WHO IV | 291 | 38.85 |
| PRS_type | Primary | 502 | 67.02 |
|  | Recurrent | 222 | 29.64 |
|  | Secondary | 25 | 3.34 |
| Radio_status | Yes | 625 | 83.44 |
|  | No | 124 | 16.56 |
| Chemo_status | Yes | 520 | 69.43 |
|  | No | 229 | 30.57 |
| Histology | Astrocytoma | 55 | 7.34 |
|  | Anaplastic astrocytoma | 39 | 5.21 |
|  | Anaplastic Oligodendroglioma | 22 | 2.94 |
|  | Anaplastic oligoastrocytoma | 80 | 10.68 |
|  | Glioblastoma | 176 | 23.50 |
|  | Oligodendroglioma | 35 | 4.67 |
|  | oligoastrocytoma | 95 | 12.68 |
|  | relapse astrocytoma | 20 | 2.67 |
|  | relapse Anaplastic astrocytoma | 36 | 4.81 |
|  | relapse Anaplastic Oligodendroglioma | 15 | 2.00 |
|  | relapse Anaplastic oligoastrocytoma | 48 | 6.41 |
|  | relapse Oligodendroglioma | 90 | 12.02 |
|  | relapse Oligodendroglioma | 4 | 0.53 |
|  | relapse oligoastrocytoma | 9 | 1.20 |
|  | Secondary relapse Oligodendroglioma | 25 | 3.34 |
| IDH_mutation_status | Mutant | 410 | 54.74 |
|  | Wildtype | 339 | 45.26 |
| 1p19q_codeletion_status | Codel | 155 | 20.69 |
|  | Non-codel | 594 | 79.31 |
