# Supplementary material for: CKAP2L, as an Independent Risk Factor, Closely Related to the Prognosis of Glioma
Source: Biomed Res Int. 2021 Sep 28;2021:5486131. doi: 10.1155/2021/5486131 (PMC8494202; doi:10.1155/2021/5486131)
Supplement: Supplementary 4 — Table S4: characteristics of patients with glioma based on clinical patients. [file 5486131.f4.docx]

Table S4. Characteristics of patients with glioma based on clinical patients

| Characteristics |  | Number of cases | Percentages(%) |
| --- | --- | --- | --- |
| Gender | Male | 21 | 52.5 |
|  | Female | 19 | 47.5 |
| Age | <=60 | 19 | 47.5 |
|  | >60 | 21 | 52.5 |
| Grade | WHO III | 17 | 42.5 |
|  | WHO IV | 23 | 57.5 |
| PRS_type | Primary | 40 | 100 |
| Chemo_status | Yes | 12 | 30 |
|  | No | 28 | 70 |
| Preoperative KPS score | 50 | 2 | 5 |
|  | 60 | 9 | 22.5 |
|  | 70 | 19 | 47.5 |
|  | 80 | 9 | 22.5 |
|  | 90 | 1 | 2.5 |
